# Inner Ear Therapeutics: An Overview of Middle Ear Delivery

**DOI:** 10.3389/fncel.2019.00261

**Published:** 2019-06-11

**Authors:** Jaimin Patel, Mikhaylo Szczupak, Suhrud Rajguru, Carey Balaban, Michael E. Hoffer

**Affiliations:** ^1^Department of Otolaryngology, University of Miami Miller School of Medicine, Miami, FL, United States; ^2^Department of Biomedical Engineering, University of Miami, Coral Gables, FL, United States; ^3^Department of Otolaryngology and Biomedical Engineering, University of Pittsburgh, Pittsburgh, PA, United States; ^4^Department of Neurological Surgery, University of Miami Miller School of Medicine, Miami, FL, United States

**Keywords:** transtympanic, intratympanic, middle ear, cochlea, labyrinthine

## Abstract

There are a variety of methods to access the inner ear and many of these methods depend on utilizing the middle ear as a portal. In this approach the middle ear can be used as a passive receptacle, as part of an active drug delivery system, or simply as the most convenient way to access the inner ear directly in human subjects. The purpose of this volume is to examine some of the more cutting-edge approaches to treating the middle ear. Before considering these therapies, this manuscript provides an overview of some therapies that have been delivered through the middle ear both in the past and at the current time. This manuscript also serves as a review of many of the methods for accessing the inner ear that directly utilize or pass though the middle ear. This manuscript provides the reader a basis for understanding middle ear delivery, the basis of delivery of medicines via cochlear implants, and examines the novel approach of using hypothermia as a method of altering the responses of the inner ear to damage.

## Introduction/Overview

Accessing the inner ear can be challenging due to its location and because direct access to the cochlea can result in hearing loss and/or balance disorders. As such, investigators and clinicians have attempted to access the inner ear via the middle ear since it is easier to reach both though the tympanic membrane (in the clinic) or through the middle ear itself (via a surgical approach). Using the middle ear as a route to the inner ear, however, still allows for a plethora of approaches to the inner ear itself. In this chapter we highlight some of the most important approaches the middle ear affords you when accessing the inner ear.

Initially we examine a technique that places an active substance in the middle ear to produce the desired effect in the inner ear. This is by far the most common type of approach to the inner ear and can be accomplished by flooding the middle ear with medication by simply injecting the fluid into the middle ear through the tympanic membrane in clinic. The nomenclature for this approach can be confusing since it appears as transtympanic, intratympanic, middle ear delivery, and a variety of other jargon. For the purposes of this overview chapter we will use the terminology “transtympanic” to refer to the procedure where a needle is passed through the tympanic membrane and allowed to fill the middle ear with medication. This approach can be used to treat a variety of disorders with different medications (only a few are highlighted in this overview chapter). A number of additional tweaks to this technique have been employed to obtain greater distribution of medication to the inner ear. These include delivery devices (some of which have appeared and been abandoned over time and others of which are just emerging onto the market), integrating the substance of interest into a biodegradable compound that is injected or surgically placed into the middle ear to slowly release doses over time. With some variation, all of these achieve the same goal of getting the active ingredient in contact with the inner ear. All of these approaches, again, can be used for an array of disorders and with an assortment of compounds. Finally, pharmaceuticals are not the only modality that can impact the inner ear. Our lab is examining how to utilize the middle ear to deliver therapeutic hypothermia to the inner ear as a countermeasure against inner ear trauma from a variety of sources. This chapter provides an overview of therapies that utilize the middle ear as a highway to the inner ear, which are the fundamental pathways for understanding how to best treat inner ear disorders.

## Transtympanic Drug Therapy

The anatomical configuration of the ear can be separated into three components: external, middle and inner ear. Pathologies arising in the inner ear are difficult to treat due to anatomical and systemic barriers. Anatomically, the inner ear is housed in the petrous bone, one of the densest bones of the human body, with limited access through the orifice of the external auditory canal. Systemically, the blood-labyrinthine barrier limits the delivery of medicine to the inner ear, similar to that of the blood brain barrier (Glueckert et al., 2018).

Transtympanic injection was first described in 1879 by Liel as a method for treating Eustachian Tube Dysfunction (Liel, 1879). The technique has been used for a variety of conditions over last 150 years and continues to be one of the most accessible routes for delivering local therapeutics to the inner ear (Mader et al., 2018). To better understand the mechanism of how this occurs, we have to address factors in both the middle and the inner ear. The middle ear is composed of the tympanic cavity with the ossicles as well as the Eustachian tube that drains into the nasopharynx. For the purpose of inner ear medication delivery, the critical portions of the middle ear are the oval window and the round window. When therapeutics are injected through the tympanic membrane (or through a hole in the tympanic membrane) the middle ear serves as the reservoir for the drug. The inner ear houses the organ of hearing, the cochlea, and the organs of balance, the vestibule and the semicircular canals (El Kechai et al., 2015). The cochlea is intricately connected to the middle ear via the two natural fenestrations of the perilympathic space: the oval window and the round window. These openings act in concert to allow fluid in the inner ear to propagate and allow audition to occur. These two windows and their associated membranes are gateways to make drug therapy into the inner ear possible (Glueckert et al., 2018) and allow the medications to reach both the hearing and balance organs of the inner ear.

## Round Window Membrane Permeability

The round window membrane (RWM) in humans consists of three layers: (1) an outer epithelial layer facing the middle ear continuous with the promontory; (2) a middle connective tissue layer; (3) an inner cellular layer that interfaces with the scala tympani (ST). Despite the three layers, studies have shown that the round window behaves as if it is semi-permeable. The epithelium of the outer layer has characteristic microvilli which are indicative of absorptive capabilities of the RWM. In addition, the inner layer lacks continuity in the basement membrane with loose junctions suggesting open passages for substances to transverse the tertiary layer into the ST. In addition, the inner layer has shown to contain pinocytotic vesicles with amorphous substances, possibly perilymph, suggesting an active role in the transfer of substances between the RWM and the ST (Goycoolea and Lundman, 1997). Moreover, detailed anatomic observations of the RWM indicates that the epithelia of the outer and inner layer are metabolically active. They both contain secretory granules, endoplasmic reticulum, and golgi within in the cells supporting a bifunctional purpose of absorption and/or secretion (Richardson et al., 1971; Miriszlai et al., 1978; Salt et al., 2012). Therefore, the RWM is an ideal gateway to deliver drugs into the inner ear.

The factors that influence the permeability of the RWM include the molecule’s size, liposolubility, electrical charge and the thickness of the membrane, to list a few. Therefore, substances that are smaller in molecular size, higher in liposolubility and positive in electrical charge will more easily diffuse into the ST via the RWM (Swan et al., 2008). Of note, transport of negatively charged nanoparticles has been reported (Youm et al., 2016). Additionally, the RWM has shown to be highly sensitive to manipulation in an effort to increase the diffusion rate of substances. Mikulec et al. (2008) demonstrated that the RWM permeability can be increased through introducing dry suctioning near the membrane along with preservatives such as benzyl alcohols and increasing osmolality of the substance. Other experiments altering the permeability of the RWM included use of local anesthetics, endotoxins, exotoxins, and histamine (Salt and Plontke, 2009). These external forces do not cause permanent damage to the RWM, but all impact the outer layer, likely making it the key layer in facilitating substances through the RWM (Goycoolea, 2001). The exact mechanism of how transmembrane transport continues to be incompletely understood. Our limited knowledge suggests that passive diffusion, facilitated diffusion through carriers, active transport, and phagocytosis may all play a role in the transmigration of substances across the RWM. This suggests that the mode of transport may be specific to the properties of each respective molecule (Salt and Plontke, 2009). It is important to remember that access to the round window can be affected by many factors that include adhesions, mucoperiosteal folds, and bone dust. Moreover the RWM itself can be thickened (Crane et al., 2005). All of these factors affect the ability of medicinews to reach the inner ear.

## Oval Window

As the oval window is covered by the stapes footplate, less investigation has been done on the oval window as a route of delivering drugs. Studies have shown that the oval window may play a role in facilitating inner ear diffusion of medication, however, quantification of the entry remains problematic since the bony footplate can impedes pure oval window delivery mechanisms (Mikulec et al., 2009; Salt et al., 2012; King et al., 2013).

## Drug Distribution in the Inner Ear

Following entry into the ST, the next challenge is to ensure distribution of the drug within the inner ear. This geometrically intricate structure has large fluid filled extracellular spaces, termed scalae, containing the inner ear fluid, perilymph. Perilymph is ionically similar to that of fluids in other extracellular spaces. The two large spaces containing perilymph include scala tympani (ST) and scala vestibuli (SV). The two scalae run parallel to one another in the spiral fashion of the cochlea, interconnected at the helicotrema, the cochlear apex. Between the two large scalae lies the scala media (SM), which contains the cellular machinery for audition. The scala media houses the endolymph, a unique fluid, high in potassium. Endolymph provides the ideal environment to transduce mechanical motion into electrical potential by the hair cells and their supporting structures (Swan et al., 2008).

The process of drug distribution has been subdivided into “radial” (with transport through the modiolus, the central core of the cochlea), and “longitudinal” (as if the cochlear spiral is unwound and fluid is flowing in a linear fashion between the parallel scalae and connected at the helicotrema) (Ohyama et al., 1988). One example of radial distribution was shown in 1991 by Salt et al. (1991) in which substances were found in the highest concentration at the basal turn and at the vestibular system after transmigrating the RWM. The mechanism behind this was believed to be that substances traveled between the extracellular spaces via the spiral ligament in the modiolus. With respect to longitudinal distribution, a number of factors come into play before a substance can theoretically achieve uniform distribution. In contrast to other extracellular fluids, the perilymph does not flow or is not actively stirred (Ohyama et al., 1988; Salt and Plontke, 2005). As a result, the drug distribution is slow and dependent on passive diffusion. The rate at which a molecule will passively diffuse in perilymph is dependent upon the diffusion coefficient, as the perilymph is in essence stagnant. A number of physical properties determine the diffusion coefficient of a molecule. Studies have shown that molecular weight is the most important factor (Salt, 2005). Another crucial aspect to drug distribution is clearance of the drug, which is the removal of a substance from the extracellular space into the capillary beds or the modiolus, metabolized in the perilymph or bound with tissue. Therefore, the relationship between a drug’s diffusion capacity and clearance rate is key to determining its distributive quality. Consequently, to design an evenly distributed drug, the ideal configuration is a small molecule that is cleared slowly (Salt and Ma, 2001). A number of different medications and modalities have been engineered to ensure adequate delivery of medication to the inner ear.

## Transtympanic Treatment Modalities

Transtympanic injections are the simplest approach to delivering medicine to the inner ear, however, not the most efficient. This method has a number of variables unaccounted for, such as the clearances of the solution by the Eustachian tube, prolonged direct contact with the RWM, and effective transport through the RWM. Different drug delivery modalities and devices have been developed to overcome these challenges. The two well studied devices include the Silverstein Microwick^®^and the Microcather (*μCat*^®^). The Silverstein Microwick^®^is an absorbable polyvinyl acetate wick that is inserted through a myringotomy and placed overlying the round window. The myringotomy is kept open with a ventilation tube allowing the patient to instill the drug solution through the external auditory canal themselves. The solution is absorbed by the wick, reducing Eustachian tube clearance, and ensures direct contact with the RWM for effective passive diffusion (Silverstein et al., 2004). The Microcather (*μCat*^®^) is designed with an external and internal compartment. The external end contains biluminal ports, one for infusion and the other for withdrawal of fluid, while the internal end has a inflatable bulbous tip. This device is inserted after a tympanomeatal flap has been raised, where the bulbous tip is place within the round window niche and the biluminal end exits out of the external auditory canal. This end is connected to various pumping systems for drug infusions such as micropumps and osmotic pumps (Swan et al., 2008). This system provided more control of the amount of medicine instilled into the area of the round window but was associated with a risk of hearing loss in some centers (Thomsen et al., 2000) Osmotic pumps have been used to test a number of therapies for many inner ear disease in animal models and has been reviewed by Pararas et al. (2012).

The previous two devices do require exposing the middle ear for treatment making them more invasive. So forth, research in developing different injectable solutions was done to overcome the barriers of the middle ear. One such solution is hydrogels. Hydrogels are solutions with high viscosity and unique properties that allow environmental triggers to release drugs into the surrounding area (Mader et al., 2018). The viscous nature of the solution helps reduce Eustachian tube clearance, consequently increasing the residence time in the middle ear. This in turn supports increased RMW exposure time. For example, Poloxamer 407 is a hydrogel that is temperature sensitive. At room temperature it is an injectable liquid, but once it resides at body temperature within the middle ear, it gelifies allowing adequate drug exposure time (Wang et al., 2009). These drug-hydrogel solutions have been used both clinically and in animal studies (El Kechai et al., 2015). Recently, one has completed two Phase 3 clinical trials. The company Otonomy has created OTIVIDEX, a sustained-exposure formulation of transtympanic injectable dexamethasone for the treatment of Meniere’s disease. This drug-hydrogel has shown to have significant benefits in patients with Meniere’s disease (Mader et al., 2018).

Nanoparticular injection systems have also garnered attention recently for otologic purposes. These nanocarriers are of interest due to their ability to permeate the RWM and deliver their payload to targeted tissues. Some well-studied nanocarriers include liposomes, superparamagnetic iron oxide nanoparticles (SPIONs), and PLGA nanoparticles to name a few (Ge et al., 2007; Pritz et al., 2013; Bozzuto and Molinari, 2015). Liposomes are small phospholipid bilayer structures with an aqueous core. Due to their bilayer property, a liposome can encapsulate both hydrophobic and hydrophilic substances. This helps facilitate transport of substances through the RWM efficiently, while being able carry either type of substance. In addition, they allow surface modification with PEG, antibodies, peptides, carbohydrates, chitosan, hyaluronic acid and folic acid (Bozzuto and Molinari, 2015). Poly (lactic-co-glycolic acid) (PLGA) nanoparticles are biodegradable polymers versatile and safe enough for parenteral administration (Kumari et al., 2010). Different PLGA polymers have different properties allowing them to encapsulate hydrophobic and hydrophilic molecules while allowing surface modification with PEGylation, antibody ligands and chitosan adsorption (Grottkau et al., 2013). PLGA nanoparticle’s adaptability and diversity in modification makes them an interesting drug delivery system for inner ear disease. SPIONs are Fe_3_O_4_ particles that are magnetized by an external magnetic field to control the migration of particles through the RWM. These particles do not encapsulate molecules; therefore, they contain a polymeric layer where PLGA nanoparticles are bound carrying the drug payload (Ge et al., 2007). This is a novel delivery mechanism where we can magnetically control the delivery of medication into the inner ear. Through continued research, a number of different nanoparticles, hydrogels, and substances are being engineered to ensure adequate and targeted therapy to the inner ear with the safe, non-invasive approach of transtympanic injections.

## Transtympanic Use of Medication

A variety of drugs have been used to tackle some of the common diseases that affect the inner ear. Some examples include corticosteroids, aminoglycoside antibiotics, antioxidants, anesthetics, and neurotrophins. Even vectors for gene therapy have been introduced through transtympanic injections (Kanzaki, 2018). The most common transtympanic drugs includes corticosteroids and aminoglycosides as they treat a number of common diseases, such as sudden sensorineural hearing loss (SSNHL), tinnitus, and Meniere’s disease. Therefore, these two classes of medications will be discussed in detail.

As a medication class, steroids are known for their immunosuppressive qualities and in this case, electrolyte altering properties as well (Fukushima et al., 2002). The exact mechanism of action is still elusive but a number of known properties of this class of agent likely play a role in the ear as well and these include the following: 1) suppression of irritability or hypersensitivity of the sensory cells in the inner ear; 2) reduction of immune-mediated inflammation/autoimmune dysfunction; and/or 3) direct effect on the inner ear neuroepithelium (Yilmaz et al., 2005). SSNHL is debilitating to patients and steroids are the first line of treatment. Despite a paucity of high level evidence supporting the use of steroids for SSNHL, oral steroids are the standard of care for this disorder. A number of studies have suggested that oral steroids are equally as effective as transtympanic steroids, however, there are side effects associated with long term use of oral steroids (Hong et al., 2009; Dispenza et al., 2011; Rauch et al., 2011; Filipo et al., 2013). Additionally, transtympanic delivery can achieve a 100-fold higher concentration of steroids in the perilymph versus systemic delivery without the severe side effects (Bird et al., 2007). Clinical trials combining systemic and transtympanic versus transtympanic alone has shown to have no significant difference (Tsounis et al., 2018). So forth, there continues to be variations in an exact protocol on how to treat SSNHL. There are discrepancies on the frequency of injections, treatment length, and the ideal steroid of choice. Ultimately, the clinician’s judgment about the choice of therapy should be patient centered, airing on the side of risk profile and cost efficiency (Stachler et al., 2012; Bear and Mikulec, 2014).

In recent years there have been five prospective, randomized, controlled trials with blinding, investigating the use of transtympanic steroids for the treatment of tinnitus. For the three studies comparing transtympanic steroid infusion to saline, there was not a statistically significant difference between the two groups with both producing a placebo-like improvement (Araujo et al., 2005; Topak et al., 2009; Choi et al., 2013). Another study that compared transtympanic dexamethasone or prednisolone to oral carbamazepine, showed no difference in tinnitus control rates among the groups (She et al., 2009). The remaining study, which only included patients that developed symptoms of tinnitus within the previous 3 months, was the only one to demonstrate a statistically significant difference between the two groups that received transtympanic steroids compared to the group that did not (25.8% and 25.0% vs. 9.8%, *p* < 0.05) (Shim et al., 2011). These works are not without limitations such as the low concentrations of dexamethasone employed (Araujo et al., 2005; Choi et al., 2013) as well as the small sample sizes (Chandrasekhar, 2014). The latter is inherent to studies of this design as it is difficult to conduct randomized, controlled trials with the number of patients Sakata et al. (1996) did near the end of the 20th century.

Meniere’s disease (MD) causes devastating vertigo attacks with roaring tinnitus and aural fullness. As the disease progresses hearing loss is also inevitable (Syed et al., 2015). An algorithm on treatment of MD has evolved over time (Nevoux et al., 2018). Up to eighty percent of patients are either cured or in remission especially from vertigo from first line therapy, which includes lifestyle change, low salt diet, and diuretics (Claes and Van de Heyning, 2000). The remaining patients are treated conservatively with transtympanic steroids. Improvement in MD with steroids has been hypothesized to occur if hydrops is due to inflammation or autoimmune factors (Foster, 2015). Transtympanic steroids have been shown to improve both frequency and severity of vertigo spells compared to placebo at 24 months after treatment (Nevoux et al., 2018). Patel et al. (2016) reports two injections of methylprednisolone given 2 weeks apart is effective as gentamicin for the treatment of refractory MD, without the ototoxicity (Patel et al., 2016). Following the treatment protocol, refractory MD is treated with local destructive medical treatment with transtympanic gentamicin (TTG). However, gentamicin is not without risk. TTG is recommended if hearing function is already impaired as it is severely ototoxic. Based on a meta-analysis by Syed et al. (2015), a “titration” protocol of TTG (40 mg/ml) until disappearance of vertigo has been described (Syed et al., 2015). This protocol is in an effort to preserve hearing through directed therapy over exposing the patient to systemic gentamicin.

## Intracochlear Treatment

The next step in inner ear therapy is direct delivery to the root of the problem. Intracochlear administration is delivering the drug directly to the cochlea thus avoiding the middle ear barriers, such as the RWM and the Eustachian tube. While this modality involves direct inner ear administration the middle ear is usually the route to access the inner ear. The general modalities of introducing intracochlear medications includes direct injection, osmotic pumps, a constant infusion system, cochlear implant coating, and microfluidic reciprocating reservoir (El Kechai et al., 2015). As of this date, not all of these have been translated into human use.

Clinically, intracochlear injections are only possible using a surgical approach. Access is established through a cochleostomy either through the RWM or through the basal turn of the cochlea where a needle is inserted, and the drug is delivered. This method’s inherent problem is the possibility of a leak at the injection site. Experiments have been done to control the fluid efflux with internal and external sealing procedures which show promising results (Plontke et al., 2016). An osmotic pump utilizes the osmotic gradient between the cannister containing the drug and the perilymph to drive the drug through the cannula and into the cochlea at a rate determined by the device. A number of drugs and gene vectors have been delivered through this mechanism in animal models (Borenstein, 2011).

The constant infusion system employs two points of entry, one in the cochlea and one in the posterior semicircular canal. The cochleostomy is at the basal turn and has an infusion pump through a cannula which supplies the drug at a predetermined rate. The inherent problem of a leak is threat with this technique. The canalostomy, near the SV, provides a fluid outlet to reduce concentration gradient within the perilymph enhancing, drug diffusion to the apex (Borkholder et al., 2010). An additional hinderance to this delivery method is the low rate of clearance of cochlear fluid, hence a limited volume of drug can be introduced through the pump in a given time span. To address this limitation, the reciprocating microfluidic reservoir was developed to provide a net zero volume delivery system that infused and withdrew a constant volume of drug in a cyclical fashion. Strategically, the infusion portion of the cycle lasts a few seconds and a total drug concentration in 1 μL is delivered. This fluid mixture is cycled through the cochlea through a withdraw port and into the device to be cycled through the infusion port in a cyclical fashion. This ensures adequate mixture and delivery by controlling the variables of flow and clearance rate of the cochlear fluid (Tandon et al., 2016).

The most logical method of intracochlear drug administration is through a cochlear implant. Patients that can qualify for this invasive procedure have profound hearing loss making it an ideal situation to implant a device directly into the inner ear. This device is placed surgically through a cochleostomy or through the RWM. This convenient situation allows the opportunity to deliver drugs to patients for a number of different applications. Some possible pathways include coating the device in biodegradable eluting polymers or integrating an active infusion pump within the device (Borenstein, 2011). One current effort to utilize this opportunity is to reduce the histological trauma of inserting the implant (Eshraghi et al., 2005). Different experiments have been conducted to reduce the inflammation and fibrosis of tissue, most applying the coating capabilities of the device with biodegradable polymers. El Kechai et al. (2015) has reviewed these thoroughly. With regards to an active pump integration, cochlear implant catheters have been combined with intracochlear implants in animal models, up to 15 mm of insertion, to test delivering a single bolus of iodine concurrently with the insertion of the device (Ibrahim et al., 2011). This method shows promise with delivery of iodine without leak or radiologic damage to the inner ear. However, further work needs to be done to determine the efficacy between different drugs and the quantity of the bolus injectable without damaging the delicate structures of the inner ear.

## Recent Advances

Recent advances in novel inner ear therapeutics include different drug modalities, the robust application of genetic manipulation through viral vectors, and even hypothermic inner ear treatments. One particular study shows the inhibition of cochlear N-methyl-D-aspartate (NMDA) receptors with AM-101, a small novel antagonist, to treat tinnitus triggered by glutamate excitotoxicity. Clinical trials with this inhibitor showed that 3 transtympanic injections over 3 consecutive days of 0.81 mg/ml of AM-101 demonstrated a significant and dose dependent improvement in tinnitus (Staecker et al., 2015). N-Acetylcysteine (NAC), a low molecular weight agent with significant otoprotective qualities has also been tested clinically. Transtympanic injections have been studied with this agent to combat the ototoxic side effects of cisplatin, a well-known phenomenon from this chemotherapeutic agent. A 10% NAC solution was injected transtympanically prior to the infusion of cisplatin versus the control and the pure tone audiometry was measured. The study concluded that control patients had a significant reduction at 8000 HZ showing that NAC may play a role in otoprotection against ototoxic elements (Riga et al., 2013). With regards to autoimmune inner ear disease, a clinical trial with transtympanic TNF-alpha inhibitor (infliximab) was shown to improve hearing and reduce disease relapse (Van Wijk et al., 2006). As the next frontier of medicine is genetic manipulation, the inner ear is an ideal location to utilize this technology. Kanzaki (2018) has thoroughly reviewed the literature with regards to the application of viral vectors targeting the inner ear. Predominantly this technology is still in its early phase with animal models. However, as further studies are conducted, the ideal vision is to allow Otolaryngologists the ability to deliver targeted therapy to different types of inner ear cells based on the pathological presentation of the patient. Furthermore, as previously mentioned in the intracochlear treatment section, cochlear implant insertion can cause significant damage to the inner ear, particularly loss of residual hearing. Tamames et al. (2016) has created an animal model to test a custom probe that is infused with cooled fluorocarbon adjacent to the middle turn of the cochlea prior to implant insertion, as a means to protect the inner ear. Their work has demonstrated that rats with normothermic cochlea had significant loss of residual hearing compared to the hypothermic group due to implant trauma. Histologic studies confirmed the findings with significant loss of outer hair cells in the normothermic group. They showed this method to be feasible in human temporal bones with the cochlea cooling down 4 to 6 °C with their custom designed probe. This novel method to combat implant insertion trauma is promising to further our efforts of inner ear therapeutics.

## Conclusion

Inner ear therapeutics are undergoing tremendous progress with a wide range of drug selection and different delivery methods. The anatomical intricacies of the ear employ a difficult challenge to drug delivery, however, through the advancements of knowledge and technology we have been able to locally deliver medication to the inner ear. Transtympanic injections are relatively simple procedures performed routinely by Otolaryngologists. This simple procedure has a complex physiological mechanism that is not yet fully understood. However, through further experimentation and novel pharmaceutical design the goal of treating inner ear disease is possible. As our understanding of the oval window, RWM, inner ear pharmacokinetics, drug distribution and mechanisms continue to grow, so will our applicability to better patient outcomes. With more invasive procedures such as intracochlear therapies, the development of miniaturized devices as a means to deploy therapies and even cooling the cochlea are promising ventures. With the future of medicine being targeted therapies, genetics may be the next avenue in clinical trials for inner ear disease. However, with our current understanding, further research is necessary to address the pharmaceutical and physiological challenges to safely and efficaciously treat all inner ear diseases.

## Author Contributions

JP prepared the manuscript. MS collected the data and prepared the manuscript. SR collected the data and edited the manuscript. CB edited the manuscript. MH prepared the manuscript and edited the manuscript.

## Conflict of Interest Statement

The authors declare that the research was conducted in the absence of any commercial or financial relationships that could be construed as a potential conflict of interest.

## References

[B1] Araujo M. F. Oliveira C. A. Bahmad F. M. Jr. (2005). Intratympanic dexamethasone injections as a treatment for severe, disabling tinnitus: does it work? *Arch. Otolaryngol. Head Neck Surg.* 131 113–117. 15723941 10.1001/archotol.131.2.113

[B2] Bear Z. W. Mikulec A. A. (2014). Intratympanic steroid therapy for treatment of idiopathic sudden sensorineural hearing loss. *Mol. Med.* 111 352–356. PMC6179477 25211869

[B3] Bird P. A. Begg E. J. Zhang M. Keast A. T. Murray D. P. Balkany T. J. (2007). Intratympanic versus intravenous delivery of methylprednisolone to cochlear perilymph. *Otol. Neurotol.* 28 1124–1130. 10.1097/mao.0b013e31815aee21 18043438

[B4] Borenstein J. T. (2011). Intracochlear drug delivery systems. *Expert. Opin. Drug Deliv.* 8 1161–1174. 10.1517/17425247.2011.588207 21615213 PMC3159727

[B5] Borkholder D. A. Zhu X. Hyatt B. T. Archilla A. S. Livingston W. J. III Frisina R. D. (2010). Murine intracochlear drug delivery: reducing concentration gradients within the cochlea. *Hear. Res.* 268 2–11. 10.1016/j.heares.2010.04.014 20451593 PMC2933796

[B6] Bozzuto G. Molinari A. (2015). Liposomes as nanomedical devices. *Int. J. Nanomed.* 10 975–999. 10.2147/IJN.S68861 25678787 PMC4324542

[B7] Chandrasekhar S. S. (2014). In reference to intratympanic dexamethasone injection for refractory tinnitus: prospective placebo-controlled study. *Laryngoscope* 124:E255. 24089258 10.1002/lary.24438

[B8] Choi S. J. Lee J. B. Lim H. J. In S. M. Kim J. Y. Bae K. H. (2013). Intratympanic dexamethasone injection for refractory tinnitus: prospective placebo-controlled study. *Laryngoscope* 123 2817–2822. 10.1002/lary.24126 23620150

[B9] Claes J. Van de Heyning P. H. (2000). A review of medical treatment for Meniere’s disease. *Acta Otolaryngol. Suppl.* 544 34–39. 10904799 10.1080/000164800750044461

[B10] Crane B. T. Minor L. B. Della Santina C. C. Carey J. P. (2005). Middle ear exploration in patients with Ménière’s disease who have failed outpatient intratympanic gentamicin therapy.Otol Neurotol 2009; 30: 619-24; Penha, R and Escada,P. Round-window anatomical considerations in intratympanic drug therapy for inner-ear diseases. *Int. Tinnitus J.* 11 31–33. 19503016 10.1097/MAO.0b013e3181a66d2b

[B11] Dispenza F. Amodio E. De Stefano A. Gallina S. Marchese D. Mathur N. (2011). Treatment of sudden sensorineural hearing loss with transtympanic injection of steroids as single therapy: a randomized clinical study. *Eur. Arch. Otorhinolaryngol.* 268 1273–1278. 10.1007/s00405-011-1523-0 21327728

[B12] El Kechai N. Agnely F. Mamelle E. Nguyen Y. Ferrary E. Bochot A. (2015). Recent advances in local drug delivery to the inner ear. *Int. J. Pharm.* 494 83–101. 10.1016/j.ijpharm.2015.08.015 26260230

[B13] Eshraghi A. A. Polak M. He J. Telischi F. F. Balkany T. J. Van De Water T. R. (2005). Pattern of hearing loss in a rat model of cochlear implantation trauma. *Otol. Neurotol.* 26 442–447; discussion 447. 15891647 10.1097/01.mao.0000169791.53201.e1

[B14] Filipo R. Attanasio G. Russo F. Y. Viccaro M. Mancini P. Covelli E. (2013). Intratympanic steroid therapy in moderate sudden hearing loss: a randomized, triple-blind, placebo-controlled trial. *Laryngoscope* 123 774–778. 10.1002/lary.23678 23378346

[B15] Foster C. A. (2015). Optimal management of Meniere’s disease. *Ther. Clin. Risk Manag.* 11 301–307. 10.2147/TCRM.S59023 25750534 PMC4348125

[B16] Fukushima M. Kitahara T. Uno Y. Fuse Y. Doi K. Kubo T. (2002). Effects of intratympanic injection of steroids on changes in rat inner ear aquaporin expression. *Acta Otolaryngol.* 122 600–606. 10.1080/000164802320396268 12403121

[B17] Ge X. Jackson R. L. Liu J. Harper E. A. Hoffer M. E. Wassel R. A. (2007). Distribution of PLGA nanoparticles in chinchilla cochleae. *Otolaryngol. Head Neck Surg.* 137 619–623. 10.1016/j.otohns.2007.04.013 17903580

[B18] Glueckert R. Johnson Chacko L. Rask-Andersen H. Liu W. Handschuh S. Schrott-Fischer A. (2018). Anatomical basis of drug delivery to the inner ear. *Hear. Res.* 368 10–27. 10.1016/j.heares.2018.06.017 30442227

[B19] Goycoolea M. V. (2001). Clinical aspects of round window membrane permeability under normal and pathological conditions. *Acta Otolaryngol.* 121 437–447. 10.1080/000164801300366552 11508501

[B20] Goycoolea M. V. Lundman L. (1997). Round window membrane. structure function and permeability: a review. *Microsc. Res. Tech.* 36 201–211. 10.1002/(sici)1097-0029(19970201)36:3<201::aid-jemt8>3.0.co;2-r 9080410

[B21] Grottkau B. E. Cai X. Wang J. Yang X. Lin Y. (2013). Polymeric nanoparticles for a drug delivery system. *Curr. Drug Metab.* 14 840–846. 10.2174/138920021131400105 24016112

[B22] Hong S. M. Park C. H. Lee J. H. (2009). Hearing outcomes of daily intratympanic dexamethasone alone as a primary treatment modality for ISSHL. *Otolaryngol. Head Neck Surg.* 141 579–583. 10.1016/j.otohns.2009.08.009 19861194

[B23] Ibrahim H. N. Bossard D. Jolly C. Truy E. (2011). Radiologic study of a disposable drug delivery intracochlear catheter. *Otol. Neurotol.* 32 217–222. 10.1097/MAO.0b013e3182040c47 21178805

[B24] Kanzaki S. (2018). Gene delivery into the inner ear and its clinical implications for hearing and balance. *Molecules* 23:E2507. 10.3390/molecules23102507 30274337 PMC6222543

[B25] King E. B. Salt A. N. Kel G. E. Eastwood H. T. O’Leary S. J. (2013). Gentamicin administration on the stapes footplate causes greater hearing loss and vestibulotoxicity than round window administration in guinea pigs. *Hear. Res.* 304 159–166. 10.1016/j.heares.2013.07.013 23899413 PMC3800104

[B26] Kumari A. Yadav S. K. Yadav S. C. (2010). Biodegradable polymeric nanoparticles based drug delivery systems. *Colloids Surf. B Biointerf.* 75 1–18. 10.1016/j.colsurfb.2009.09.001 19782542

[B27] Liel W. (1879). On intratympanic injections in catarrhal affections of the middle ear. *Br. Med. J.* 2:364. 10.1136/bmj.2.975.364 20749291 PMC2240732

[B28] Mader K. Lehner E. Liebau A. Plontke S. K. (2018). Controlled drug release to the inner ear: concepts, materials, mechanisms, and performance. *Hear. Res.* 368 49–66. 10.1016/j.heares.2018.03.006 29576310

[B29] Mikulec A. A. Hartsock J. J. Salt A. N. (2008). Permeability of the round window membrane is influenced by the composition of applied drug solutions and by common surgical procedures. *Otol. Neurotol.* 29 1020–1026. 10.1097/MAO.0b013e31818658ea 18758387 PMC2596477

[B30] Mikulec A. A. Plontke S. K. Hartsock J. J. Salt A. N. (2009). Entry of substances into perilymph through the bone of the otic capsule after intratympanic applications in guinea pigs: implications for local drug delivery in humans. *Otol. Neurotol.* 30 131–138. 10.1097/mao.0b013e318191bff8 19180674 PMC2729139

[B31] Miriszlai E. Benedeczky I. Csapo S. Bodanszky H. (1978). The ultrastructure of the round window membrane of the cat. *ORL J. Otorhinolaryngol. Relat. Spec.* 40 111–119. 10.1159/000275393 570687

[B32] Nevoux J. Barbara M. Dornhoffer J. Gibson W. Kitahara T. Darrouzet V. (2018). International consensus (ICON) on treatment of Meniere’s disease. *Eur. Ann. Otorhinolaryngol. Head Neck Dis.* 135 S29–S32. 10.1016/j.anorl.2017.12.006 29338942

[B33] Ohyama K. Salt A. N. Thalmann R. (1988). Volume flow rate of perilymph in the guinea-pig cochlea. *Hear. Res.* 35 119–129. 10.1016/0378-5955(88)90111-6 3198505

[B34] Pararas E. E. Borkholder D. A. Borenstein J. T. (2012). Microsystems technologies for drug delivery to the inner ear. *Adv. Drug Deliv. Rev.* 64 1650–1660. 10.1016/j.addr.2012.02.004 22386561 PMC3387506

[B35] Patel M. Agarwal K. Arshad Q. Hariri M. Rea P. Seemungal B. M. (2016). Intratympanic methylprednisolone versus gentamicin in patients with unilateral Meniere’s disease: a randomised, double-blind, comparative effectiveness trial. *Lancet* 388 2753–2762. 10.1016/S0140-6736(16)31461-1 27865535

[B36] Plontke S. K. Hartsock J. J. Gill R. M. Salt A. N. (2016). Intracochlear drug injections through the round window membrane: measures to improve drug retention. *Audiol. Neurootol.* 21 72–79. 10.1159/000442514 26905306 PMC4842307

[B37] Pritz C. O. Dudas J. Rask-Andersen H. Schrott-Fischer A. Glueckert R. (2013). Nanomedicine strategies for drug delivery to the ear. *Nanomedicine* 8 1155–1172. 10.2217/nnm.13.104 23837855

[B38] Rauch S. D. Halpin C. F. Antonelli P. J. Babu S. Carey J. P. Gantz B. J. (2011). Oral vs intratympanic corticosteroid therapy for idiopathic sudden sensorineural hearing loss: a randomized trial. *JAMA* 305 2071–2079. 10.1001/jama.2011.679 21610239

[B39] Richardson T. L. Ishiyama E. Keels E. W. (1971). Submicroscopic studies of the round window membrane. *Acta Otolaryngol.* 71 9–21. 10.3109/00016487109125327 5541454

[B40] Riga M. G. Chelis L. Kakolyris S. Papadopoulos S. Stathakidou S. Chamalidou E. (2013). Transtympanic injections of N-acetylcysteine for the prevention of cisplatin-induced ototoxicity: a feasible method with promising efficacy. *Am. J. Clin. Oncol.* 36 1–6. 10.1097/COC.0b013e31822e006d 22134515

[B41] Sakata E. Itoh A. Itoh Y. (1996). Treatment of cochlear-tinnitus with dexamethasone infusion into the tympanic cavity. *Int. Tinnitus J.* 2 129–135. 10753351

[B42] Salt A. N. (2005). Pharmacokinetics of drug entry into cochlear fluids. *Volta. Rev.* 105 277–298. 17330152 PMC1805693

[B43] Salt A. N. King E. B. Hartsock J. J. Gill R. M. O’Leary S. J. (2012). Marker entry into vestibular perilymph via the stapes following applications to the round window niche of guinea pigs. *Hear. Res.* 283 14–23. 10.1016/j.heares.2011.11.012 22178981 PMC3277668

[B44] Salt A. N. Ma Y. (2001). Quantification of solute entry into cochlear perilymph through the round window membrane. *Hear. Res.* 154 88–97. 10.1016/s0378-5955(01)00223-4 11423219

[B45] Salt A. N. Ohyama K. Thalmann R. (1991). Radial communication between the perilymphatic scalae of the cochlea. II: estimation by bolus injection of tracer into the sealed cochlea. *Hear. Res.* 56 37–43. 10.1016/0378-5955(91)90151-x 1769923

[B46] Salt A. N. Plontke S. K. (2005). Local inner-ear drug delivery and pharmacokinetics. *Drug Discov. Today* 10 1299–1306. 10.1016/s1359-6446(05)03574-9 16214674 PMC2681268

[B47] Salt A. N. Plontke S. K. (2009). Principles of local drug delivery to the inner ear. *Audiol. Neurootol.* 14 350–360. 10.1159/000241892 19923805 PMC2820328

[B48] She W. Dai Y. Du X. Chen F. Ding X. Cui X. (2009). Treatment of subjective tinnitus: a comparative clinical study of intratympanic steroid injection vs. *oral carbamazepine*. *Med. Sci. Monit.* 15 I35–I39. 19478715

[B49] Shim H. J. Song S. J. Choi A. Y. Hyung Lee R. Yoon S. W. (2011). Comparison of various treatment modalities for acute tinnitus. *Laryngoscope* 121 2619–2625. 10.1002/lary.22350 22109762

[B50] Silverstein H. Thompson J. Rosenberg S. I. Brown N. Light J. (2004). Silverstein microWick. *Otolaryngol. Clin. North Am.* 37 1019–1034. 10.1016/j.otc.2004.04.002 15474108

[B51] Stachler R. J. Chandrasekhar S. S. Archer S. M. Rosenfeld R. M. Schwartz S. R. Barrs D. M. (2012). Clinical practice guideline: sudden hearing loss. *Otolaryngol.Head Neck Surg.* 146 S1–S35. 22383545 10.1177/0194599812436449

[B52] Staecker H. Maxwell K. S. Morris J. R. van de Heyning P. Morawski K. Reintjes F. (2015). Selecting appropriate dose regimens for AM-101 in the intratympanic treatment of acute inner ear tinnitus. *Audiol. Neurootol.* 20 172–182. 10.1159/000369608 25872149

[B53] Swan E. E. Mescher M. J. Sewell W. F. Tao S. L. Borenstein J. T. (2008). Inner ear drug delivery for auditory applications. *Adv. Drug Deliv. Rev* 60 1583–1599. 10.1016/j.addr.2008.08.001 18848590 PMC2657604

[B54] Syed M. I. Ilan O. Nassar J. Rutka J. A. (2015). Intratympanic therapy in Meniere’s syndrome or disease: up to date evidence for clinical practice. *Clin. Otolaryngol.* 40 682–690. 10.1111/coa.12449 25916787

[B55] Tamames I. King C. Bas E. Dietrich W. D. Telischi F. Rajguru S. M. (2016). A cool approach to reducing electrode-induced trauma: localized therapeutic hypothermia conserves residual hearing in cochlear implantation. *Hear. Res.* 339 32–39. 10.1016/j.heares.2016.05.015 27260269 PMC5018430

[B56] Tandon V. Kang W. S. Robbins T. A. Spencer A. J. Kim E. S. McKenna M. J. (2016). Microfabricated reciprocating micropump for intracochlear drug delivery with integrated drug/fluid storage and electronically controlled dosing. *Lab. Chip* 16 829–846. 10.1039/c5lc01396h 26778829 PMC4766044

[B57] Thomsen J. Charabi S. Tos M. (2000). Preliminary results of a new delivery system for gentamicin to the inner ear in patients with Meniere’s disease. *Eur. Arch. Otorhinolaryngol.* 257 362–365. 10.1007/s004059900219 11052245

[B58] Topak M. Sahin-Yilmaz A. Ozdoganoglu T. Yilmaz H. B. Ozbay M. Kulekci M. (2009). Intratympanic methylprednisolone injections for subjective tinnitus. *J. Laryngol. Otol.* 123 1221–1225. 10.1017/S0022215109990685 19640315

[B59] Tsounis M. Psillas G. Tsalighopoulos M. Vital V. Maroudias N. Markou K. (2018). Systemic, intratympanic and combined administration of steroids for sudden hearing loss. a prospective randomized multicenter trial. *Eur. Arch. Otorhinolaryngol.* 275 103–110. 10.1007/s00405-017-4803-5 29168028

[B60] Van Wijk F. Staecker H. Keithley E. Lefebvre P. P. (2006). Local perfusion of the tumor necrosis factor alpha blocker infliximab to the inner ear improves autoimmune neurosensory hearing loss. *Audiol. Neurootol.* 11 357–365. 10.1159/000095897 16988499

[B61] Wang X. Dellamary L. Fernandez R. Harrop A. Keithley E. M. Harris J. P. (2009). Dose-dependent sustained release of dexamethasone in inner ear cochlear fluids using a novel local delivery approach. *Audiol. Neurootol.* 14 393–401. 10.1159/000241896 19923809

[B62] Yilmaz I. Yilmazer C. Erkan A. N. Aslan S. G. Ozluoglu L. N. (2005). Intratympanic dexamethasone injection effects on transient-evoked otoacoustic emission. *Am. J. Otolaryngol.* 26 113–117. 10.1016/j.amjoto.2004.11.001 15742264

[B63] Youm I. Musazzi U. M. Gratton M. A. Murowchick J. B. Youan B. C. (2016). Label-free ferrocene-loaded nanocarrier engineering for in vivo cochlear drug delivery and imaging. *J. Pharm. Sci.* 105 3162–3171. 10.1016/j.xphs.2016.04.012 27449230

